# Life’s Essential 8 and Depression in the U.S.: Findings From National Health and Nutrition Examination Survey

**DOI:** 10.1016/j.focus.2026.100537

**Published:** 2026-07-27

**Authors:** Abraham M. Enyeji, Andrew D. Roland, Jayson Forbes, David Wetzell

**Affiliations:** 1 Mercer University School of Medicine, Columbus, Georgia; 2 Public Health Department, Kiran C. Patel College of Osteopathic Medicine, Nova Southeastern University, Fort Lauderdale, Florida; 3 Department of Economics, Michigan State University, East Lansing, Michigan

**Keywords:** Life’s Essential 8, cardiovascular health, depression, NHANES, depressive symptoms, mental health

## Abstract

**Introduction:**

Cardiovascular health and mental health are closely interconnected; however, the relationship between the American Heart Association’s updated Life’s Essential 8 cardiovascular health metric and depression severity has not been fully characterized in U.S. adults. The association between Life’s Essential 8 and depressive symptom severity was examined in a nationally representative sample of U.S. adults.

**Methods:**

Cross-sectional data from the National Health and Nutrition Examination Survey were analyzed. Life’s Essential 8 scores were calculated using behavioral (smoking, diet, physical activity, sleep health, and BMI) and health factor (blood pressure, cholesterol, and HbA1c) components. Depressive symptoms were assessed using the Patient Health Questionnaire-9 and categorized as no/minimal (scores <10), moderate (scores 10–18), and severe (scores ≥19) depression. Survey-weighted multinomial logistic regression models were used to estimate ORs and 95% CIs for moderate and severe depression relative to those for no/minimal depression after adjustment for sociodemographic covariates.

**Results:**

Higher Life’s Essential 8 scores were associated with lower odds of moderate depression, with each 1-point increase corresponding to approximately 0.4% lower odds (OR per 1-point increase=0.996; 95% CI=0.994, 0.998; *p*=0.001), whereas no significant association was observed for severe depression (OR=0.996; 95% CI=0.988, 1.003; *p*=0.230). Among individual Life’s Essential 8 components, favorable smoking status and healthy sleep duration demonstrated the strongest inverse associations with moderate depression, whereas healthy sleep duration was also associated with lower odds of severe depression. Male gender and higher income-to-poverty ratios were associated with lower odds of moderate depression, whereas single marital status was associated with higher odds.

**Conclusions:**

Higher cardiovascular health, as measured by Life’s Essential 8, was associated with lower odds of moderate but not severe depression among U.S. adults. Behavioral cardiovascular health indicators, particularly smoking status and sleep health, showed the strongest associations with depressive symptom severity. These findings support the broader relevance of cardiovascular health in relation to mental health and highlight the need for longitudinal studies to clarify temporal and potentially bidirectional relationships between Life’s Essential 8 metrics and depression severity.

## HIGHLIGHTS

• Higher Life’s Essential 8 scores were associated with lower odds of moderate depression.
• No significant association was observed between Life’s Essential 8 and severe depression.
• The association between cardiovascular health and depression varied by severity.
• Sleep health and smoking were strongly associated with depression status.
• Findings support links between cardiovascular and mental health in U.S. adults.

## INTRODUCTION

Depression is a common and debilitating mental health disorder characterized by persistent sadness, low mood, loss of interest, cognitive difficulties, and impaired daily functioning. It remains a leading contributor to disability worldwide and is associated with substantial personal and societal burden.^1^, ^2^, ^3^ In the U.S., depression affects millions of adults annually and is associated with increased healthcare utilization; reduced productivity; and elevated risk of chronic medical conditions, including cardiovascular disease (CVD).

The American Heart Association (AHA) introduced the concept of ideal cardiovascular health (CVH) to promote cardiovascular well-being and reduce the burden of CVD. This framework was recently expanded with Life’s Essential 8 (LE8), a comprehensive metric for assessing CVH across the lifespan.^4^ LE8 evaluates 4 health behaviors—diet quality, physical activity, nicotine exposure, and sleep health—and 4 health factors, including normalized BMI, blood cholesterol, blood glucose (HbA1c), and blood pressure. Together, these 8 components provide a quantitative measure of CVH that reflects both lifestyle behaviors and physiologic health status.

Evidence indicates that higher LE8 scores are associated with favorable cardiovascular outcomes, including lower risk of cardiovascular events, reduced mortality, and longer life expectancy.^4^^,^^5^ Individuals with ideal LE8 metrics demonstrate healthier profiles across multiple domains, underscoring the importance of lifestyle and risk factor management in preventing disease. Beyond cardiovascular outcomes, emerging research suggests that the behavioral and health factors captured by LE8 may also influence mental health. Greater leisure time physical activity has been consistently associated with a lower likelihood of depressive symptoms, supporting its role as a key behavioral component of CVH. Emerging evidence further suggests that physical activity is associated with healthier dietary patterns, including lower dietary inflammatory potential and higher consumption of foods containing live microbes. These dietary factors may partially mediate the relationship between physical activity and depression, highlighting interconnected behavioral pathways within the LE8 framework.^6^^,^^7^ Consistent with this framework, recent studies using nationally representative U.S. data have reported inverse associations between overall LE8 scores and depressive symptoms, with preliminary evidence suggesting dose response relationships.^8^

In addition, a broader body of evidence indicates that lifestyle behaviors, including physical inactivity, tobacco use, unhealthy dietary patterns, and inadequate sleep, are consistently associated with a higher prevalence and greater severity of depressive symptoms in adults.^9^, ^10^, ^11^

Similarly, CVH factors included in LE8, such as elevated BMI, hypertension, dysglycemia, and abnormal lipid profiles, have been linked to depression through shared biological pathways, including systemic inflammation, dysregulation of stress response systems, and vascular dysfunction.^12^, ^13^, ^14^ Behavioral exposures such as smoking and diets high in processed foods may further increase depressive symptoms through neuroinflammatory and metabolic mechanisms.

Recent studies using nationally representative U.S. data have reported inverse associations between LE8 and depressive symptoms, with preliminary evidence of dose response relationships.^15^, ^16^, ^17^ Although prior studies have strengthened the understanding of age-specific, gender-specific, and methodologic variations in the LE8 depression relationship, less is known about whether CVH metrics demonstrate differential associations across clinically relevant gradients of depressive symptom severity. Examining moderate and severe depression separately may provide additional insight into whether CVH indicators are more strongly associated with earlier or less severe depressive states than with more complex depressive phenotypes.

Similar associations have also been demonstrated across diverse methodologic and population-based frameworks. For example, Jin et al.^18^ and Liu and colleagues^19^ reported consistent inverse relationships between overall LE8 scores and depressive symptom burden in nationally representative cohorts. More specialized analyses have further suggested that individual behavioral components of LE8 may differentially contribute to mental health outcomes. In emerging adults, sleep health and physical activity were identified as major contributors to the association between LE8 and depressive symptoms.^8^ Recent work has additionally incorporated machine learning approaches to better characterize nonlinear relationships between CVH metrics and depression risk, whereas gender-specific analyses have demonstrated potential heterogeneity in these associations across men and women.^19^

Prior literature demonstrates that psychosocial risk factors operate across the full spectrum of depression severity but exert stronger effects in severe depression.^20^^,^^21^ In addition, different stressor domains may differentially predict moderate versus severe depression, with more severe forms being linked to higher cumulative and qualitatively distinct stress exposures.^22^ Indeed, cumulative stress exposure has been identified as a key determinant of severe depressive phenotypes, underscoring the importance of examining whether associations with CVH vary by depression severity.

To address this gap, this study used data from the National Health and Nutrition Examination Survey (NHANES) to examine the association between LE8 and depression severity, focusing on moderate and severe depression among U.S. adults. Using multinomial logistic regression, the study evaluated whether these associations differed across levels of depression severity, enabling assessment of both the overall relationship between LE8 and depression and the extent to which individual CVH components were differentially associated with moderate versus severe depressive symptoms.

## METHODS

### Study Sample

This study used cross sectional secondary data from the NHANES collected between 2011 and 2018. NHANES is a nationally representative survey designed to assess the health and nutritional status of the civilian, non-institutionalized U.S. population. The survey uses a complex, multistage probability sampling design to obtain a representative sample of U.S. residents and collects both self-reported and objectively measured health information. Data include demographic characteristics, health behaviors, dietary intake, physical examination measures, and laboratory biomarkers. Detailed descriptions of NHANES methods have been published previously.^23^ The analytic sample included adults aged ≥20 years who participated in the 2011–2018 cycles of the NHANES. Participants were eligible for inclusion regardless of depression status. The final study sample comprised 3,165 individuals after combining multiple NHANES data sets containing relevant variables from demographic, questionnaire, and dietary data files.

### Measures

History of depression was determined using self-reported information collected during the NHANES interview. Depressive symptoms were assessed using Patient Health Questionnaire 9 (PHQ 9), a validated self-report screening instrument widely used to measure the severity of depressive symptoms in population-based studies. The PHQ 9 consists of 9 items corresponding to the diagnostic criteria for major depressive disorder, each scored from 0 to 3, yielding a total score ranging from 0 to 27. Consistent with established thresholds, participants with a PHQ 9 score of 10 or greater were classified as having clinically significant depressive symptoms, whereas those with scores below 10 were categorized as not having depression or mild forms of it.^24^ For analyses examining depression severity, PHQ 9 scores were further categorized into 3 groups: minimal/mild (≤9), moderate (10–18), and severe (>18), consistent with established severity thresholds, with categories collapsed to facilitate statistical modeling.

LE8 was introduced by the AHA in 2022 as an updated framework for evaluating CVH, building upon the earlier Life’s Simple 7 construct.^25^ The revised framework expands the original model by incorporating sleep health and by applying updated scoring methods designed to more precisely quantify CVH status.

Each component is scored on a standardized scale ranging from 0 to 100, allowing CVH to be evaluated as a continuous measure with greater sensitivity than earlier categorical approaches. For this analysis, individual metric scores were calculated according to established LE8 algorithms and averaged to generate an overall CVH score. The resulting composite score was subsequently classified into 3 categories reflecting CVH status: poor (≤49), intermediate (50–79), and ideal (≥80), consistent with previously published applications of the LE8 framework.^4^^,^^26^^,^^27^

During study visits, participants completed standardized, self-administered questionnaires designed to capture multiple aspects of their lifestyle behaviors. Dietary intake was assessed using the Automated Self-Administered 24 hour Dietary Assessment Tool, in which participants documented all foods and beverages consumed within 24 hours immediately preceding the assessment, limited to the first study day. Diet quality was subsequently quantified using the Healthy Eating Index 2015 (HEI 2015), a 13-component scoring system ranging from 0 to 100, with higher scores reflecting closer adherence to recommended dietary guidelines. For this investigation, HEI 2015 scores derived from the initial 24-hour recall were used as the primary indicator of overall dietary pattern quality.^28^ The index comprises diverse nutritional domains, including total and whole fruit intake, total vegetables, legumes, dairy, whole grains, plant proteins, total protein foods, seafood, fatty acid ratios, refined grains, sodium, and calories from added sugars and saturated fats. Participants were classified according to HEI 2015 thresholds as having poor diet quality (≤50), intermediate quality (51–80), or ideal diet quality (>80). Standardized nutrient and food group variables for computing several HEI 2015 components were obtained from the Food Patterns Equivalents Database and NHANES total nutrient files, including measures of fruit, dairy, whole grains, refined grains, added sugars, sodium, and saturated fat (Table A.5, available online).

Physical activity was evaluated using a 3-day reporting framework, allowing recorded activity durations to be converted into weekly totals of moderate-to-vigorous physical activity. Sleep health was captured through self-reported habitual sleep duration.^8^^,^^29^ Tobacco exposure was defined according to the AHA’s nicotine exposure criteria and determined by assessing participants’ current, former, or never smoking status as well as exposure to secondhand smoke through cohabitation with regular smokers (Table A.4, available online).^30^

Health factors were assessed following standardized NHANES protocols. BMI (kg/m²) was calculated by dividing measured body weight (kg) by the square of standing height (m²). Baseline systolic and diastolic blood pressure values were determined as the means of all measurements taken during the initial study visit. Serum lipid concentrations were measured using enzymatic assays, and non–high-density lipoprotein cholesterol was derived by subtracting high-density lipoprotein cholesterol from total cholesterol. HbA1c levels were determined using high-performance liquid chromatography. Comprehensive descriptions of each CVH metric, including the scoring criteria, are provided in the Appendix Materials (available online) and have been extensively detailed in prior research.^31^

The outcome measure, the prevalence of depression, was assessed using a self-administered questionnaire based on the 9-item PHQ 9. In this analysis, PHQ 9 depression severity scores were categorized into minimal/no depression (PHQ 9 ≤9), moderate depression (10–18), and severe depression (>18), consistent with established PHQ 9 severity thresholds, with categories collapsed to facilitate statistical modeling.^24^ Participants reported the frequency of depressive symptoms experienced over the preceding 2 weeks, with each item scored from 0 (not at all) to 3 (nearly every day). Total PHQ 9 scores ranged from 0 to 27, with higher scores indicating greater symptom severity. Consistent with established cut offs, scores ≥10 were used to define the presence of clinically significant depressive symptoms, corresponding to moderate to severe depression.

In terms of covariates, according to previous NHANES literature,^8^^,^^29^ demographic data were collected during in home interviews, including participant reported age, gender, race, ethnicity, educational level, marital status, and annual household income. For analytical purposes, participants were categorized into 3 age groups: 20–39, 40–59, and 60–79 years. Race and ethnicity were self-reported following NHANES standards and classified as non-Hispanic (NH) Asian, NH Black, NH White, Mexican American, or multiracial.

Family income level was measured using a monthly poverty index in which participants reported their total household income from the previous month in dollars. This measure reflected the ratio between the reported monthly income and the established poverty threshold. Household poverty status was determined by comparing monthly family income with the poverty guidelines established by the HHS. Income categories included very low income (<1.0), low income (1.01–2.0), lower middle income (2.01–3.0), middle income (3.01–4.0), and high income (>4.0).

### Statistical Analysis

Descriptive statistics were used to summarize the characteristics of the study population, with participants stratified by depression severity. All analyses incorporated NHANES complex survey design variables, including sampling weights, strata, and primary sampling units, in accordance with National Center for Health Statistics analytic guidelines. Survey-weighted means and proportions were calculated to generate nationally representative estimates. Continuous variables are reported as means with 95% CIs, whereas categorical variables are presented as unweighted counts and survey weighted percentages. Differences in baseline CVH measures across depression severity groups were assessed using 1-way ANOVA for continuous variables and Rao-Scott chi-square tests for categorical variables.

The association between LE8 scores and prevalent depression by severity level was examined using multinomial logistic regression models. Regression coefficients (β) were exponentiated to obtain ORs and corresponding 95% CIs, using the standard transformation OR = exp(β). All LE8-related estimates are presented as AORs reflecting the change in odds of depressive symptoms per unit increase in LE8 score or across predefined CVH categories.

In addition, multinomial logistic regression models were used as the primary severity-based analysis to evaluate whether associations between LE8 and depression differed across severity levels. In these models, participants with no or minimal depressive symptoms (PHQ 9 <10) served as the reference category, and AORs were estimated for moderate (PHQ 9 10–18) and severe (PHQ 9 >18) depression, allowing assessment of differential associations across the depression severity spectrum.

In a prespecified subanalysis, LE8 scores were disaggregated into their individual components (health behaviors and health factors) to evaluate their independent associations with depression severity. Each component was modeled separately within multinomial logistic regression frameworks to determine its contribution to the overall association between LE8 and depression across severity categories.

This study was reviewed and approved by the Mercer University IRB (H26 02001). Because the analysis involved secondary analysis of deidentified, person-level data, it was determined to be exempt from human subject review. All data analyzed in this study are publicly available and can be accessed through the NHANES website, ensuring transparency and reproducibility.

## RESULTS

In the final analytic sample, participants were stratified into no or minimal, moderate, and severe depressive symptom groups on the basis of PHQ 9 scores. Overall, the distribution of demographic, socioeconomic, and CVH characteristics differed significantly across depression severity categories (Table A.1, available online).

A clear gender difference was observed across depression severity levels, with a lower proportion of males in the moderate and severe depression groups than in the no/minimal depression group (50.0% vs 36.9% and 43.1%, respectively; *p*<0.001). Age stratification showed a significant difference primarily in the 40–59-year group, where the proportion increased progressively with depression severity (34.6% in no/minimal, 39.3% in moderate, and 44.0% in severe depression; *p*=0.004), whereas no significant differences were observed in the youngest or oldest age groups.

Participants with household incomes below the poverty threshold (<1.0 income-to-poverty ratio) demonstrated a substantially higher representation in the moderate (29.1%) and severe (34.8%) depression groups than in the no/minimal depression subgroup (14.4%; *p*<0.001). In contrast, participants with household incomes greater than 4.01 times the poverty threshold were most prevalent in the no/minimal depression group (39.4%) and were significantly less represented in the moderate (21.5%) and severe (20.0%; *p*<0.001) depression groups. Educational attainment showed similar patterns, with college-level education or higher being significantly less common in the moderate and severe depression groups (14.9% and 15.2%, respectively) than in the no/minimal group (31.3%; *p*<0.001). Marital status was also significantly associated with depression severity, with married individuals comprising a progressively smaller proportion across increasing severity (52.9% in no/minimal vs 34.6% in moderate vs 29.0% in severe; *p*<0.001), whereas widowed/divorced/separated individuals were more represented in higher severity categories.

Across race and ethnicity, significant differences were observed for NH Asian and multiracial participants. NH Asian individuals were least represented in the moderate and severe depression groups (*p*<0.001), whereas multiracial participants showed an increasing proportion with worsening depression severity (3.3% vs 6.2% vs 8.0%; *p*=0.007). Mean LE8 scores revealed a graded inverse relationship with depression severity. Participants with no or minimal depressive symptoms had higher mean LE8 scores (70.8; 95% CI=70.0, 71.6) than those in the moderate (67.6; 95% CI=66.0, 69.1) and severe (67.4; 95% CI=61.3, 72.1; *p*=0.0004) depression subgroups.

Regarding CVH behaviors, poorer profiles were observed with increasing depression severity. Smoking prevalence was significantly higher among individuals without depressive symptoms (40.2%) than among those with moderate (24.7%) and severe (23.3%; *p*<0.001) depression. Sleep health also worsened progressively across severity categories (77.7% vs 64.4% vs 57.9%; *p*<0.001). Physical activity and diet quality did not differ significantly across groups.

Among CVH factors, hypertension, cholesterol, and diet quality did not differ significantly across depression severity categories. In contrast, HbA1c showed a borderline association with depression severity (*p*=0.056), with slightly higher values observed in the moderate and severe depression groups than in the no/minimal group. A lower prevalence of normal BMI was also observed in the moderate and severe depression groups than in the no/minimal group (58.0% and 60.1% vs 65.7%, respectively; *p*<0.001).

In multivariable multinomial logistic regression analyses, each 1-unit increase in LE8 score was associated with a 0.4% reduction in the odds of moderate depression (OR=0.996 95% CI=0.994, 0.998; *p*=0.001). A similar but nonsignificant association was observed for severe depression (OR=0.996; 95% CI=0.988, 1.003; *p*=0.230), indicating a graded inverse relationship that was more pronounced for moderate depressive symptoms (Table A.2, available online).

SES demonstrated strong and consistent associations with depression severity. Individuals with incomes between 2.01 and 3.0 times the poverty threshold had significantly lower odds of both moderate (OR=0.484; 95% CI=0.286, 0.819; *p*=0.009) and severe (OR=0.194; 95% CI=0.052, 0.715; *p*=0.016) depression than those whose household income was below the poverty threshold (<1.0 income-to-poverty ratio). Similarly, individuals with household incomes greater than 4.01 times the poverty threshold demonstrated lower odds of moderate depression (OR=0.486; 95% CI=0.290, 0.814; *p*=0.008), whereas the association with severe depression approached statistical significance (OR=0.416; 95% CI=0.163, 1.066; *p*=0.066). Gender differences were evident, with males exhibiting lower odds of moderate depression than females (OR=0.635; 95% CI=0.428, 0.941; *p*=0.025), although no significant association was observed for severe depression. Age was not significantly associated with either moderate or severe depression after adjustment.

Regarding sociodemographic characteristics, being single was strongly associated with higher odds of moderate depression (OR=2.363; 95% CI=1.480, 3.774; *p*=0.001), with a weaker and nonsignificant association observed for severe depression. Marital disruption (widowed/divorced/separated) and educational attainment were not consistently associated with depression severity after adjustment. Race and ethnicity showed limited associations overall; however, NH Asian participants had significantly lower odds of severe depression than NH White participants.

Table A.3 (available online) and the corresponding forest plots below present the AORs for moderate and severe depression according to individual LE8 components and covariates. Overall, higher LE8 scores were associated with lower odds of moderate depression, whereas associations with severe depression were less consistent.

Among LE8 components, several behavioral factors were found to be inversely associations with depression severity. Favorable smoking status was associated with lower odds of moderate depression (OR=0.37; 95% CI=0.21, 0.63), whereas sleep health was inversely associated with both moderate (OR=0.39; 95% CI=0.19, 0.81) and severe (OR=0.20, 95% CI=0.05, 0.78) depression. BMI showed a similar inverse trend for moderate depression (OR=0.55, 95% CI=0.30, 1.01), although the association was not statistically significant for severe depression. Other components, including physical activity, HbA1c, cholesterol, hypertension, and diet quality, showed no consistent or statistically significant associations with either moderate or severe depression. Overall, behavioral components of LE8, particularly smoking and sleep, showed the most consistent relationships with depression, with stronger and more stable associations observed for moderate depression than for severe depression.

In contrast, other LE8 components, including diet quality, physical activity, cholesterol, hypertension, and HbA1c, showed weaker or no clear associations with depression. These findings suggest that behavioral CVH factors may play a particularly important role in the relationship between CVH and depression. Behavioral health metrics represent modifiable lifestyle factors that influence both cardiometabolic and psychological pathways, including inflammation, neuroendocrine regulation, and health behaviors linked to mood and well-being. Prior research has similarly demonstrated that unhealthy behaviors such as smoking, poor sleep, and obesity are strongly associated with increased depression risk, supporting the role of behavioral CVH as a shared pathway linking mental and cardiometabolic health.^4^^,^^31^^,^^32^

In survey-weighted multinomial logistic regression analyses, higher overall LE8 scores were associated with lower odds of moderate depression than of no/minimal depression (OR=0.996; 95% CI=0.994, 0.998; *p*=0.001), whereas no significant association was observed for severe depression (OR=0.996; 95% CI=0.988, 1.003; *p*=0.230) (Table A.2, available online). Among the individual LE8 components, favorable smoking status and healthy sleep duration demonstrated the strongest associations with lower odds of moderate depression. Participants meeting optimal smoking metrics had a 63% lower likelihood of moderate depression (OR=0.37; 95% CI=0.21, 0.63; *p*=0.001), whereas healthy sleep duration was associated with significantly lower odds of both moderate (OR=0.39; 95% CI=0.19, 0.81; *p*=0.013) and severe (OR=0.20; 95% CI=0.05, 0.78; *p*=0.022) depression. Other LE8 components, including physical activity, diet, blood pressure, cholesterol, HbA1c, and BMI, were not independently associated with depression severity after multivariable adjustment.

Several sociodemographic variables were also independently associated with depression severity. Male gender was associated with lower odds of moderate depression (OR=0.63; 95% CI=0.42, 0.95; *p*=0.029), whereas single marital status was associated with more than a twofold increase in the odds of moderate depression (OR=2.22; 95% CI=1.37, 3.61; *p*=0.002). SES demonstrated a graded inverse association with depressive symptom severity. Participants with income levels between 2.01 and 3.0 times the poverty threshold had significantly lower odds of moderate (OR=0.53; 95% CI=0.31, 0.90; *p*=0.021) and severe (OR=0.22; 95% CI=0.06, 0.77; *p*=0.020) depression than those whose household income fell below the poverty threshold (<1.0 income-to-poverty ratio). Similarly, participants with household incomes >4.01 times the poverty threshold had lower odds of moderate depression (OR=0.52; 95% CI=0.31, 0.88; *p*=0.017), with a similar but nonsignificant trend observed for severe depression (OR=0.45; 95% CI=0.17, 1.15; *p*=0.092). Race and ethnicity were not significantly associated with moderate or severe depression in adjusted analyses, with the exception of NH Asian participants, who demonstrated substantially lower odds of severe depression than the reference group (*p*<0.001). Age was not independently associated with depression severity after adjustment for clinical and socioeconomic covariates. Prior studies have suggested that lower observed rates of severe depression among older adults may, in part, reflect survival bias associated with the increased mortality risk accompanying severe depressive illness.^21^

Figure 1 displays the AORs and 95% CIs for individual LE8 components in relation to moderate and severe depression. For moderate depression, favorable CVH metrics, including lower BMI, lower HbA1c, greater physical activity, adequate sleep duration, and nonsmoking status, were generally associated with reduced odds of depression, whereas poorer diet quality and hypertension were associated with increased odds. Similar directional associations were observed for severe depression; however, CIs were generally wider, and several estimates crossed the null value, indicating less precise associations. Overall, the findings suggest that healthier LE8-related behaviors and risk factors are more consistently associated with lower odds of moderate than severe depression among U.S. adults.

**Figure 1:**
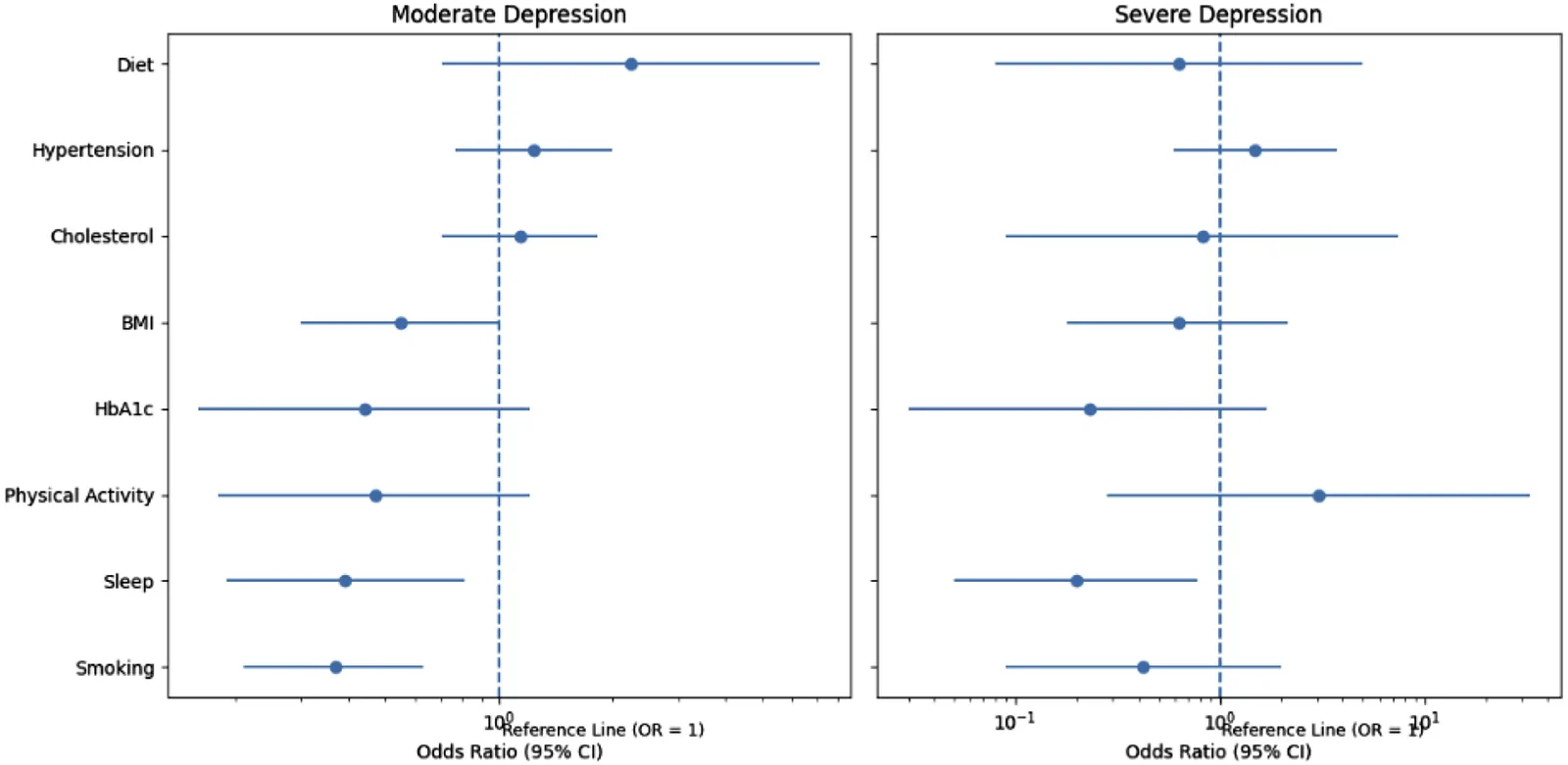
Forest Plot of adjusted odds ratios for depression by Life’s Essential 8 components among U.S. adults.

In multivariable logistic regression analyses (Appendix Table A, available online), higher LE8 scores were independently associated with lower odds of moderate depression (β= −0.0037; 95% CI= −0.0058, −0.0017; *p*=0.001), whereas no significant association was observed with severe depression (β= −0.0045; 95% CI= −0.0120, 0.0030; *p*=0.230). Participants with household income-to-poverty ratios of 2.01–3.0 and >4.01 had significantly lower odds of moderate depression than the reference income group, whereas those with ratios of 1.01–2.0 and 2.01–3.0 had significantly lower odds of severe depression. Male gender was associated with lower odds of moderate depression (β= −0.4549, 95% CI= −0.8489, −0.0608; *p*=0.025), whereas being single was associated with higher odds of moderate depression (β=0.8600, 95% CI=0.3918, 1.3282; *p*=0.001). No significant associations were observed for age, educational attainment, or most racial/ethnic groups, although NH Asian participants had substantially lower odds of severe depression than the reference group (*p*<0.001).

## DISCUSSION

This study adds to the growing body of evidence supporting an association between CVH and depression. The observed relationship between LE8 and depressive symptoms suggests that CVH may serve as an integrated marker of underlying physiologic and behavioral pathways relevant to mental health. Although cardiovascular prevention strategies have traditionally focused on reducing cardiovascular morbidity and mortality, these findings highlight the broader relevance of CVH in relation to depression at the population level.

A notable aspect of these findings is the prominent contribution of behavioral CVH indicators, consistent with prior work.^14^ These findings also align with more specialized investigations demonstrating that behavioral LE8 components may contribute disproportionately to the observed relationship between CVH and depression. In particular, You et al.^15^ identified sleep health and physical activity as primary drivers of the LE8 depression association among emerging adults, supporting the importance of behavioral pathways across diverse populations. In this study, sleep health demonstrated significant associations with both moderate and severe depression, whereas smoking and normalized BMI status were most strongly associated with moderate depression, further suggesting that individual LE8 components may exhibit differential relationships depending on depression severity.

Health behaviors, including smoking status, sleep health, and physical activity, are closely linked to neurobiological and psychosocial pathways implicated in depression. Within the CVH framework, these components demonstrated consistent associations, with healthier behavioral profiles associated with lower odds of moderate depression. Smoking has been associated with alterations in neurotransmitter systems and dysregulation of stress response pathways that may contribute to depressive symptomatology.^33^ Similarly, sleep health is integral to mood regulation, with insufficient or disrupted sleep associated with alterations in circadian rhythms, inflammatory processes, and emotional functioning.^32^ In addition, obesity and adverse weight status have been associated with depression through mechanisms involving systemic inflammation, metabolic dysregulation, and psychosocial stressors.^34^ Collectively, these findings suggest behavioral pathways as shared mechanisms through which lifestyle factors may be related to both cardiovascular and mental health outcomes.

These findings are consistent with a broader conceptual framework in which cardiovascular and mental health are interrelated through bidirectional mechanisms. Depression has been associated with adverse health behaviors; lower adherence to preventive care; and physiologic alterations, including heightened inflammatory activity and autonomic dysregulation.^35^ Conversely, favorable health behaviors have been associated with better cardiovascular and psychological health profiles. These observations underscore the importance of considering cardiovascular and mental health together within broader lifestyle and behavioral frameworks.

Socioeconomic and social determinants also appear to play an important role in the relationship between CVH and depression. Factors such as financial resources, social support, and marital status influence access to health-promoting environments, patterns of healthcare utilization, and exposure to psychosocial stressors, all of which are associated with disparities in mental health outcomes.^36^ Accounting for these contextual influences is essential for developing equitable public health approaches that address both cardiovascular and mental health.

Higher CVH, as measured by LE8, was associated with lower odds of moderate but not severe depression, suggesting that the relationship between CVH and mental health may differ according to depression severity. This pattern may indicate that favorable CVH metrics are more strongly associated with earlier or less severe stages of depression, whereas severe depression likely reflects more complex and multifactorial determinants, including higher cumulative or qualitatively distinct stress exposures.^22^^,^^37^, ^38^, ^39^, ^40^ These observations may extend prior literature by suggesting that the relationship between LE8 and depression is not uniform across the depressive symptom spectrum. Whereas previous studies have primarily evaluated overall depressive symptom burden, the present findings suggest that examining severity gradients may provide additional clinical insight into how CVH metrics relate to different depressive phenotypes. This distinction may be particularly relevant given emerging evidence demonstrating gender specific associations^41^ and the use of machine-learning approaches to identify complex relationships between LE8 components and mental health outcomes. Together, these studies suggest that the association between CVH and depression is likely multifactorial and heterogeneous across demographic and clinical subgroups.

Observed differences across race and ethnicity and marital status further underscore the contribution of social determinants. Collectively, these findings support the relevance of CVH in relation to the population burden of moderate depression.

### Limitations

Several limitations should be considered. The cross-sectional nature of the data precludes conclusions regarding causality, and bidirectional relationships between depression and CVH behaviors are likely. Depression measures were based on screening tools rather than clinical diagnoses, and some health behaviors relied on self report, which may introduce measurement bias.

Despite these limitations, the study benefits from the use of nationally representative data and the application of the updated LE8 framework, which provides a comprehensive measure of CVH. These findings contribute to the evolving understanding of the relationship between CVH and broader health outcomes beyond CVD.

## CONCLUSIONS

In conclusion, this study reinforces the close interconnection between CVH and depression, highlighting that higher LE8 scores are associated with lower odds of moderate but not severe depression. Behavioral CVH indicators, including smoking, sleep, and normalized BMI, appear to play a central role, reflecting shared physiologic and psychosocial mechanisms that influence both cardiovascular and mental health. The findings also underscore the importance of considering social determinants, such as race and ethnicity and marital status, in understanding depression disparities. Taking together, these findings support the broader relevance of CVH in relation to depressive symptom severity and suggest that behavioral CVH indicators may be differentially associated across the depression severity spectrum. Further longitudinal and mechanistic studies are needed to clarify the temporal and potentially heterogeneous relationships between LE8 components and depression across demographic and clinical subgroups.

## CRediT authorship contribution statement

**Abraham M. Enyeji:** Conceptualization, Formal analysis, Data curation, Supervision, Methodology, Investigation, Writing – original draft. **Andrew D. Roland:** Methodology, Writing – original draft, Data curation, Writing – review & editing. **Jayson Forbes:** Conceptualization, Supervision, Writing – review & editing. **David Wetzell:** Conceptualization, Formal analysis, Methodology, Investigation, Writing – review & editing.

## Appendix Supplementary materials

Image, application 1

## Footnotes

Supplementary material associated with this article can be found in the online version at doi:10.1016/j.focus.2026.100537.
