# Supplementary material for: Life’s Essential 8 and Depression in the U.S.: Findings From National Health and Nutrition Examination Survey
Source: AJPM Focus. 2026 Jul 27;5(6):100537. doi: 10.1016/j.focus.2026.100537 (PMC13756792; doi:10.1016/j.focus.2026.100537)
Supplement: Supplementary file 1 [file mmc1.pdf]

Life's Essential 8 and Depression in the United States: Findings From NHANES

Authors:

Abraham M. Enyeji MD PhD, Andrew Roland MS, Jayson Forbes PhD, David L. Wetzell PhD

American Journal of Preventative Medicine-Focus (AJPM Focus).

Appendix Table 1. Summary Statistics of participants and LE8 metrics by Depression Status, NHANES 2011–2018

Variable	No or minimal Depression (95% CI)	Moderate Depression (95% CI)	Severe Depression (95% CI)	p-value
**Self-identified Gender**				
Male	0.500 (0.491–0.509)	0.369 (0.328–0.410)	0.431 (0.359–0.504)	0.0000
**Age, yrs**				
Age 20–39	0.347 (0.329–0.364)	0.340 (0.305–0.374)	0.341 (0.247–0.435)	0.9278
Age 40–59	0.346 (0.333–0.359)	0.393 (0.356–0.431)	0.440 (0.353–0.527)	0.0040
Age 60–79	0.230 (0.216–0.244)	0.201 (0.170–0.232)	0.183 (0.121–0.246)	0.1144
**Income-Poverty line**				
<1.0	0.144 (0.130–0.158)	0.291 (0.252–0.330)	0.348 (0.256–0.440)	0.0000
1.01 – 2.0	0.198 (0.185–0.210)	0.260 (0.226–0.294)	0.212 (0.133–0.290)	0.0009
2.01 – 3.0	0.146 (0.136–0.157)	0.149 (0.117–0.181)	0.123 (0.056–0.190)	0.7766
3.01 – 4.0	0.111 (0.100–0.123)	0.074 (0.053–0.095)	0.114 (0.039–0.189)	0.0025
>4.01	0.394 (0.370–0.418)	0.215 (0.179–0.250)	0.200 (0.114–0.287)	0.0000
**Education & Marital Status**				
Some college	0.313 (0.299–0.326)	0.352 (0.312–0.392)	0.325 (0.240–0.409)	0.1479
College+	0.313 (0.289–0.338)	0.149 (0.112–0.186)	0.152 (0.071–0.232)	0.0000
Married	0.529 (0.509–0.548)	0.346 (0.304–0.388)	0.290 (0.213–0.367)	0.0000
Widowed/Divorced/Separated	0.074 (0.068–0.080)	0.127 (0.106–0.147)	0.128 (0.072–0.183)	0.0000
**Mean LE8 Scores**				
LE8 Score	70.8 (70.0-71.6)	67.6 (66.0 – 69.1)	67.4 (61.3 – 72.1)	0.0004
**Race/Ethnicity**				
Non-Hispanic Black	0.112 (0.093–0.131)	0.126 (0.097–0.155)	0.112 (0.073–0.150)	0.3738

Non-Hispanic White	0.651 (0.617–0.686)	0.625 (0.567–0.683)	0.593 (0.498–0.689)	0.2767
Non-Hispanic Asian	0.055 (0.045–0.065)	0.023 (0.016–0.031)	0.012 (0.000–0.024)	0.0000
Mexican American	0.090 (0.071–0.109)	0.087 (0.058–0.115)	0.061 (0.028–0.094)	0.1384
Multiracial	0.033 (0.028–0.038)	0.062 (0.040–0.083)	0.080 (0.016–0.144)	0.0072
**Health Factors**				
BMI	0.657 (0.645–0.670)	0.580 (0.546–0.615)	0.601 (0.533–0.668)	0.0000
Hypertension	0.671 (0.661–0.681)	0.663 (0.640–0.686)	0.693 (0.637–0.750)	0.5620
Cholesterol	0.771 (0.763–0.780)	0.753 (0.727–0.778)	0.743 (0.665–0.821)	0.3329
HbA1c	0.976 (0.974–0.978)	0.963 (0.952–0.974)	0.964 (0.940–0.988)	0.0560
**Health Behaviors**				
Diet quality	0.567 (0.563–0.571)	0.564 (0.549–0.580)	0.546 (0.517–0.576)	0.3848
Smoking	0.402 (0.385–0.418)	0.247 (0.198–0.297)	0.233 (0.139–0.328)	0.0000
Sleep	0.777 (0.767–0.786)	0.644 (0.608–0.680)	0.579 (0.508–0.651)	0.0000
Physical activity	0.940 (0.935–0.946)	0.928 (0.908–0.947)	0.961 (0.935–0.987)	0.1237

Abbreviations: FPL: Federal poverty line, CI: Confidence interval, LE8: Life's Essential 8. NHANES: National Health and Nutrition Examination Survey

Appendix Table 2: Adjusted Odds Ratios for the Association Between Life's Essential 8 Score and Depression Severity Categories in U.S. Adults (NHANES)

Variable	OR (95% CI): Moderate Depression	p-value	OR (95% CI): Severe Depression	p-value
LE8 Score	0.996 (0.994–0.998)	0.001	0.996 (0.988–1.003)	0.230
**Income-Poverty line**				
1.01 – 2.0	0.955 (0.656–1.388)	0.802	0.268 (0.097–0.738)	0.013
2.01 – 3.0	0.484 (0.286–0.819)	0.009	0.194 (0.052–0.715)	0.016
3.01 – 4.0	0.719 (0.417–1.238)	0.225	1.109 (0.236–5.210)	0.893
>4.01	0.486 (0.290–0.814)	0.008	0.416 (0.163–1.066)	0.066
**Self-identified Gender**				
Male	0.635 (0.428–0.941)	0.025	0.839 (0.416–1.691)	0.612
**Age, yrs**				

Age 40–59	0.669 (0.443–1.012)	0.057	0.564 (0.220–1.445)	0.223
Age 60–79	0.823 (0.533–1.271)	0.368	0.713 (0.241–2.107)	0.528
**Education & Marital Status**				
Widowed/Divorced/Separated	1.262 (0.837–1.903)	0.257	0.893 (0.303–2.632)	0.832
Single	2.363 (1.480–3.774)	0.001	1.537 (0.658–3.592)	0.309
Some college	1.317 (0.916–1.893)	0.132	0.537 (0.191–1.507)	0.228
College+	0.871 (0.411–1.847)	0.711	1.515 (0.447–5.125)	0.492
**Race/Ethnicity**				
Non-Hispanic Black	0.699 (0.481–1.016)	0.060	1.039 (0.461–2.342)	0.925
Non-Hispanic Asian	0.493 (0.174–1.401)	0.177	~0 (≈3.0×10^{-11})	<0.001
Mexican American	0.795 (0.445–1.419)	0.424	0.837 (0.201–3.478)	0.800

Model 1: LE8: Life's Essential 8, BMI: Body Mass Index, HbA1c: Hemoglobin A1c.

Appendix Table 3. Adjusted Odds Ratios (95% Confidence Intervals) for the Association Between Disaggregated Life's Essential 8 Components and Depression Severity (NHANES)

Reference outcome: NO or Mild Depression

Variable	OR (95% CI): Moderate Depression	p-value	OR (95% CI): Severe Depression	p-value
**Components of LE8**				
Smoking	0.37 (0.21–0.63)	0.001	0.42 (0.09–1.99)	0.266
BMI	0.55 (0.30–1.01)	0.054	0.63 (0.18–2.16)	0.452
Cholesterol	1.14 (0.71–1.83)	0.565	0.83 (0.09–7.50)	0.861
Sleep	0.39 (0.19–0.81)	0.013	0.20 (0.05–0.78)	0.022
Physical Activity	0.47 (0.18–1.21)	0.113	3.05 (0.28–32.62)	0.345
HbA1c	0.44 (0.16–1.21)	0.107	0.23 (0.03–1.70)	0.144
Hypertension	1.24 (0.77–2.00)	0.360	1.48 (0.59–3.75)	0.394
Diet	2.25 (0.71–7.12)	0.161	0.63 (0.08–4.97)	0.654
**Income-Poverty line**				
1.01 – 2.0	0.99 (0.68–1.45)	0.967	0.27 (0.10–0.77)	0.015
2.01 – 3.0	0.53 (0.31–0.90)	0.021	0.22 (0.06–0.77)	0.020
3.01 – 4.0	0.81 (0.47–1.42)	0.455	1.19 (0.24–5.84)	0.827
>4.01	0.52 (0.31–0.88)	0.017	0.45 (0.17–1.15)	0.092

**Self-identified Gender**				
Male	0.63 (0.42–0.95)	0.029	0.84 (0.42–1.69)	0.608
**Age, yrs**				
age40_59	0.80 (0.50–1.27)	0.333	0.66 (0.23–1.91)	0.433
age60_79	1.05 (0.64–1.74)	0.839	0.80 (0.21–3.07)	0.740
**Education & Marital Status**				
Widowed/Divorced/Separated	1.26 (0.83–1.91)	0.261	0.88 (0.32–2.44)	0.795
Single	2.22 (1.37–3.61)	0.002	1.39 (0.65–2.99)	0.388
Some college	1.41 (0.97–2.06)	0.067	0.59 (0.21–1.62)	0.292
College+	0.94 (0.44–2.01)	0.864	1.63 (0.51–5.22)	0.394
**Race/Ethnicity**				
Non-Hispanic Black	0.65 (0.43–1.00)	0.050	0.99 (0.39–2.51)	0.987
Non-Hispanic Asian	0.52 (0.17–1.53)	0.224	~0 ($\approx 7.9\times10^{-11}$)	<0.001
Mexican American	0.77 (0.43–1.39)	0.377	0.85 (0.25–2.89)	0.785

Model 1: LE8: Life's Essential 8, BMI: Body Mass Index, HbA1c: Hemoglobin A1c.

Appendix: Supplementary file

Appendix Table 4. Definition and scoring methodology for Life's Essential 8 (LE8) score, designed for individuals aged 20 years and older

Domain	LE8 metric	*Measurement* method	Quantifying the LE8 metric (≥ 20 years of age)
Health behaviors	Sleep	*Measurement*: Self-reported average hours slept per night.<br>*Example tool for measurement*: "On average, how many hours of sleep do you typically get per night?"	Mean hours of sleep per night (x)<br>Points — Mean duration of sleep (h)<br>100 — 7≤x < 9<br>90 — 9≤x < 10<br>70 — 6≤x < 7<br>40 — 5≤x < 6; ≥ 10

		20	4≤x < 5
		0	< 4
Dietary intake	*Measurement*: Assessed using HEI-2015; dietary intake data from two 24-h dietary recalls were converted to the USDA food pattern equivalents to compute HEI-2015 scores	Percentiles of HEI-2015	
		Points	Percentile
		100	≥ 64.8th
		80	60th – 64.8th
		60	56.11th – 60th
		40	49.48th – 56.11th
		20	42.60th – 49.48th
		0	<42.16th
Exposure to nicotine	*Measurement*: Self-reported cigarette or non-cigarette tobacco product use was assessed. *Example tool for measurement*: NHANES-SMQ	Utilization of combustible tobacco products or non-cigarette tobacco product use, as well as exposure to secondhand smoke	
		Points	Status
		100	Never smoked
		75	Former smoker, quit ≥5 years
		50	Former smoker, quit ≥1 and <5 years
		25	Former smoker, quit <1 year; currently using inhaled NDS
		0	Current smoker
Physical activity (PA)	*Measurement*: Self-reported minutes of moderate or vigorous PA per week *Example tool for measurement*: NHANES PAQ-K questionnaire	Duration (min) of moderate (or greater) intensity activity per week	
		Points	Duration (min)
		100	≥ 150
		90	120–149

(Cont'd...)

Appendix Table 1. ***(Continued)***

Domain of age)	LE8 metric	*Measurement* method	Quantifying the LE8 metric (≥ 20 years	
			80	90–119
			60	60–89
			40	30–59
			20	1–29
			0	0
Health factors	BMI	*Measurement*: Body weight (kg) divided by height squared (m^2) *Example tool for measurement*: Objective measurements of height and weight	Body weight (kg) divided by height squared (m^2) Points	BMI (kg/m^2)
			100	< 25
			75	25.0–30.0
			50	30.0–35.0
			25	35.0–40.0
			0	≥ 40.0
	Total blood cholesterol	*Measurement*: Non-HDL cholesterol Example tools for measurement: Fasting or non-fasting blood sample	Enzymatically measured total cholesterol minus HDL cholesterol (mg/dL)	
			Points	Non–HDL cholesterol (mg/dL)
			100	< 130
			60	130–159
			40	160–189
			20	190–219
	BP	*Measurement*: Appropriately measured SBP and DBP<br><br>Example tools for measurement: Appropriately sized BP cuff	Appropriately measured SBP and DBP (mm Hg) Points	BP (mm Hg)
			100	SBP <120; DBP <80 (optimal)
			75	SBP: 120–129; DBP<80 (elevated)
			50	SBP: 130–139 or DBP: 80–89 (stage 1 hypertension)
			25	SBP: 140–159 or DBP: 90–99
			0	SBP≥160 or DBP ≥100
	Blood glucose	*Measurement*: FBG or casual HbA1c<br><br>Example tools for measurement: Fasting (FBG or HbA1c) or non-fasting (HbA1c) blood sample	FBG (mg/dL) or HbA1c (%)	
			Points	Status (FBG [mg/dL]/HbA1c [%])
			100	No history of diabetes and FBG <100 (or HbA1c <5.7)
			60	No diabetes and FBG: 100–125 (or HbA1c: 5.7–6.4) (prediabetes)
			40	Diabetes with HbA1c <7.0
			30	Diabetes with HbA1c: 7.0–7.9
			20	Diabetes with HbA1c: 8.0–8.9
			10	Diabetes with Hb A1c: 9.0–9.9
			0	Diabetes with HbA1c ≥10.0

Note: The contents of this table have been adapted from the original source.[1]

Abbreviations: BMI: Body mass index; BP: Blood pressure; DASH: Dietary Approaches to Stop Hypertension; DBP: Diastolic blood pressure; FBG: Fasting blood glucose; HbA1c: Hemoglobin A1c; HDL: High-density lipoprotein; HEI: Healthy Eating Index; MEPA: Mediterranean Eating Pattern for Americans; NDS: Nicotine-delivery systems; NHANES: National Health and Nutrition Examination Survey; PA: Physical activity;
PAQ-K: Physical Activity Questionnaire K; SBP: Systolic blood pressure; SMQ: Smoking assessment; USDA: United States Department of Agriculture

Appendix Tables 1 above, and Appendix Tables 2 below, provide detailed information on the derivation of the Life's Essential 8 (LE8) score used in this study. Table S1 summarizes the definitions, measurement methods, and scoring criteria for each LE8 component, including health behaviors (diet, physical activity, sleep, and nicotine exposure) and health factors (body mass index, blood pressure, blood glucose, and blood lipids). Individual metrics were scored on a scale of 0 to 100, with higher scores indicating more favorable cardiovascular health. Table S2 outlines the Healthy Eating Index–2015 components and scoring standards used to assess dietary quality. These supplementary tables facilitate interpretation of the LE8 score, and support reproducibility of the cardiovascular health assessment used in the analyses.

Appendix Table 5. The 13 dietary components and scoring criteria of the Healthy Eating Index (HEI)-2015

Intake	Component	Max score	Standard for maximum score	Standard for minimum score of zero
Adequate	Total fruits[a]	5	≥0.8 cup equiv. per 1,000 kcal	No fruits
	Whole fruits[b]	5	≥0.4 cup equiv. per 1,000 kcal	No whole fruits
	Total vegetables[c]	5	≥1.1 cup equiv. per 1,000 kcal	No vegetables
	Greens and beans[c]	5	≥0.2 cup equiv. per 1,000 kcal	No greens and beans
	Whole grains	10	≥1.5 oz equiv. per 1,000 kcal	No whole grains
	Dairy[d]	10	≥1.3 cup equiv. per 1,000 kcal	No dairy
	Total protein foods[c]	5	≥2.5 oz equiv. per 1,000 kcal	No protein foods
	Seafood and plant proteins[e]	5	≥0.8 oz equiv. per 1,000 kcal	No seafood or plant proteins
	Fatty acids[f]	10	(PUFAs+MUFAs)/SFAs ≥2.5	(PUFAs+MUFAs)/SFAs ≤1.2
Moderate	Refined grains	10	≤1.8 oz equiv. per 1,000 kcal	≥4.3 oz equiv. per 1,000 kcal
	Sodium	10	≤1.1 g per 1,000 kcal	≥2.0 g per 1,000 kcal
	Added sugars	10	≤6.5% of energy	≥26% of energy
	Saturated fats	10	≤8% of energy	≥16% of energy

Notes: Scores are allocated proportionally based on intakes ranging between the minimum and maximum thresholds; [a]includes 100% fruit juice; [b]comprises all forms, except juice; comprises legumes (peas and beans); [d]comprises all milk products, such as yogurt, cheese, fluid milk, and fortified soy beverages; [e]comprises seeds, nuts, seafood, soy products (other than beverages), and legumes (peas and beans); [f]ratio of poly - & monounsaturated fatty acids (PUFAs and MUFAs) to saturated fatty acids (SFAs); adding the maximum number of achievable points totaled to 100 points.

Reference

1. Lloyd-Jones DM, Hong Y, Labarthe D, *et al*. Defining and setting national goals for cardiovascular health promotion and disease reduction: The american heart association's strategic impact goal through 2020 and beyond. *Circulation*. 2010; 121:586-613.

   doi: 10.1161/CIRCULATIONAHA.109.192703

Appendix Table A: Multivariable Logistic Regression (Logit Coefficients) Examining the Association Between Life's Essential 8 (LE8) Metrics and Depression Among U.S. Adults, NHANES.

Variable	Moderate vs No Depression Coeff (95% CI)	p-value	Severe Depression vs No Depression (95% CI)	p-value
LE8 Scores	-0.0037 (-0.0058, -0.0017)	0.001	-0.0045 (-0.0120, 0.0030)	0.230
**Household Income (Federal Poverty level [FPL])**				

1.01 – 2.0	-0.0464 (-0.4208, 0.3280)	0.802	-1.3178 (-2.3312, -0.3043)	0.013
2.01 – 3.0	-0.7257 (-1.2521, -0.1994)	0.009	-1.6432 (-2.9507, -0.3357)	0.016
3.01 – 4.0	-0.3301 (-0.8739, 0.2138)	0.225	0.1031 (-1.4440, 1.6502)	0.893
>4.01	-0.7223 (-1.2381, -0.2064)	0.008	-0.8763 (-1.8160, 0.0634)	0.066
**Self-identified Gender**				
Male	-0.4549 (-0.8489, -0.0608)	0.025	-0.1760 (-0.8772, 0.5252)	0.612
**Age, yrs**				
Age 40–59	-0.4016 (-0.8151, 0.0120)	0.057	-0.5726 (-1.5133, 0.3681)	0.223
Age 60–79	-0.1945 (-0.6293, 0.2402)	0.368	-0.3386 (-1.4224, 0.7453)	0.528
**Education & Marital Status**				
Wid/Div/Sep	0.2326 (-0.1784, 0.6436)	0.257	-0.1135 (-1.1948, 0.9679)	0.832
Single	0.8600 (0.3918, 1.3282)	0.001	0.4298 (-0.4193, 1.2790)	0.309
Some Colleges	0.2754 (-0.0875, 0.6382)	0.132	-0.6224 (-1.6549, 0.4102)	0.228
College+	-0.1379 (-0.8897, 0.6138)	0.711	0.4151 (-0.8041, 1.6342)	0.492
**Race/Ethnicity**				
NH Black	-0.3577 (-0.7318, 0.0164)	0.060	0.0378 (-0.7753, 0.8509)	0.925
NH Asian	-0.7066 (-1.7503, 0.3372)	0.177	-24.23 (-25.0913, -23.35)	<0.001
Mexican American	-0.2299 (-0.8097, 0.3499)	0.424	-0.1782 (-1.6035, 1.2470)	0.800

Abbreviations: Wid/Div/Sep: Widowed, divorced or separated, CI: Confidence interval, LE8: Life's Essential 8. NHANES: National Health and Nutrition Examination Survey, NH: Non Hispanic.
